# Cost Description and Comparative Cost Efficiency of Post-Exposure Prophylaxis and Canine Mass Vaccination against Rabies in N’Djamena, Chad

**DOI:** 10.3389/fvets.2017.00038

**Published:** 2017-04-03

**Authors:** Rolande Mindekem, Monique Sarah Lechenne, Kemdongarti Service Naissengar, Assandi Oussiguéré, Bidjeh Kebkiba, Daugla Doumagoum Moto, Idriss Oumar Alfaroukh, Laurent Tinoanga Ouedraogo, Sahidou Salifou, Jakob Zinsstag

**Affiliations:** ^1^Centre de Support en Santé International, N’Djamena, Chad; ^2^Swiss Tropical and Public Health Institute, Basel, Switzerland; ^3^University of Basel, Basel, Switzerland; ^4^Institut de Recherches en Elevage pour le Développement, N’Djamena, Chad; ^5^Institut Régional de Santé Publique, Ouidah, Benin; ^6^Université d’Abomey Calavi, Abomey Calavi, Benin

**Keywords:** cost efficiency, One Health, Chad, rabies control and prevention, post-exposure prophylaxis

## Abstract

Rabies claims approximately 59,000 human lives annually and is a potential risk to 3.3 billion people in over 100 countries worldwide. Despite being fatal in almost 100% of cases, human rabies can be prevented by vaccinating dogs, the most common vector, and the timely administration of post-exposure prophylaxis (PEP) to exposed victims. For the control and prevention of human rabies in N’Djamena, the capital city of Chad, a free mass vaccination campaign for dogs was organized in 2012 and 2013. The campaigns were monitored by parallel studies on the incidence of canine rabies based on diagnostic testing of suspect animals and the incidence of human bite exposure recorded at selected health facilities. Based on the cost description of the campaign and the need for PEP registered in health centers, three cost scenarios were compared: cumulative cost-efficiency of (1) PEP alone, (2) dog mass vaccination and PEP, (3) dog mass vaccination, PEP, and maximal communication between human health and veterinary workers (One Health communication). Assuming ideal One Health communication, the cumulative prospective cost of dog vaccination and PEP break even with the cumulative prospective cost of PEP alone in the 10th year from the start of the calculation (2012). The cost efficiency expressed in cost per human exposure averted is much higher with canine vaccination and One Health communication than with PEP alone. As shown in other studies, our cost-effectiveness analysis highlights that canine vaccination is financially the best option for animal rabies control and rabies prevention in humans. This study also provides evidence of the beneficial effect of One Health communication. Only with close communication between the human and animal health sectors will the decrease in animal rabies incidence be translated into a decline for PEP. An efficiently applied One Health concept would largely reduce the cost of PEP in resource poor countries and should be implemented for zoonosis control in general.

## Introduction

Rabies is a viral zoonotic disease first described in Mesopotamia in 3000 B.C. Humans are infected mainly through the domestic dog (1). Once clinical signs of rabies become apparent, the outcome is nearly 100% fatal (2). Annual human deaths due to rabies are estimated at 59,000 cases worldwide, and over 29 million people are exposed and need post-exposure prophylaxis (PEP) (3). Approximately 99% of these cases occur in Africa and Asia (1, 3). Animal rabies can be controlled through immunization and population management of the reservoir species (4). Vaccination of domestic dogs, their confinement, and application of quarantine measures following importation are key prevention strategies (5). With effective control measures in place, human exposure to rabies can be drastically reduced leading to elimination of dog-mediated rabies (6).

In the United States, dog-mediated rabies was eliminated through vaccination of dogs (7), as vividly described by Tierkel et al. (8). This success story has been replicated in Latin America, where rabies transmitted by domestic dogs has been significantly reduced (9), and the same approach shows positive effect in Tanzania and Bali (10, 11). Despite ample proof of value for public health, control of rabies in the animal reservoir remains very limited in many endemic countries to date and, in the absence of dog vaccination, human rabies cases are only prevented through administration of PEP (1, 12).

The biggest challenges in Africa and Asia are free roaming dog populations, limited available resources for dog owners, limited veterinary and human health infrastructure, low disease awareness, and absence of efficient communication between the veterinary and the human health sectors (13, 14).

The absence of efficient control at the source is excessively costly in terms of disability-adjusted life years (DALY) lost due to premature death and the cost of PEP to the public and private sectors (14, 15). Economic losses also occur in the agricultural sector due to loss of livestock.

The cost of animal rabies vaccination depends on the respective context and varies from country to country (16). Cost for PEP also fluctuates greatly between countries and vaccination scheme applied (17, 18). The highest cost accrues from rabies immunoglobulin (RIG) which, according to WHO guidelines, must be injected on day 0 together with a first active vaccination dose for category 3 exposures (single or multiple transdermal bites or scratches) (1). However, in most endemic countries, RIG is not available or not affordable for victims, and PEP is limited to wound treatment and administration of anti-rabies vaccine.

In N’Djamena, Chad, rabies research studies began in 2000 and continue to date. In 2001, a pilot dog vaccination campaign showed that the societal cost for vaccination was 1,610 FCFA (2.6 USD, current exchange rate) per vaccinated animal (19).

In 2008, the incidence of human rabies was estimated at 0.7 persons per 100,000 inhabitants, using a decision tree model (20). The same study showed that over 99% of reported animal bites were inflicted by owned, but free roaming, dogs. Based on these data, a model was established, forecasting transmission of rabies within the dog population and between dogs and humans. This model proposed that the cumulative cost of a dog mass vaccination campaign combined with PEP would be less than the cumulative costs of PEP, reaching a break-even point 6 years after the start of the intervention (21). The cost effectiveness of dog vaccination combined with PEP was proposed to be higher than for an exclusive PEP approach from the fifth year onward.

The present study aims to validate and update the model predictions, through the detailed cost analysis of a citywide mass vaccination campaign carried out in 2012 and 2013 in N’Djamena. Together with data from the continuous reporting of animal rabies cases diagnosed at the rabies laboratory of the Institut de Recherche en Elevage pour le Developpement (IRED) and human bite exposures reported from selected health centers, a prospective cost effectiveness analysis between PEP alone (scenario 1) and dog vaccination with PEP (scenario 2) was done.

Following an animal bite, communication between human and animal health facilities potentially contribute to a high cost reduction for PEP, financial gains from prevented exposures, and overall number of DALY averted. To estimate the potential extent of the added value through maximal communication between veterinarians and human health workers, a third scenario was included envisioning ideal One Health communication (scenario 3).

The costs described in this article provide the basis for the planning and organization of a national mass vaccination campaign (22) and a proposed approach to lower expenses due to rabies in view of elimination of dog-mediated human rabies by 2030, as jointly outlined by WHO, FAO, OIE, the Global Alliance for Rabies Control, and the international community (23).

## Materials and Methods

### Study Site

The study took place in N’Djamena, the capital city of Chad, with a rapidly increasing population (3.3% growth rate) of approximately 1 million inhabitants in 2012 in an area of 520 km^2^. The town is divided into 10 districts and 56 quarters (24). The vaccination intervention and data collection on animal rabies incidence and incidence of human bite exposure covered the entire administrative area of the city.

### Planning and Cost Description of the Mass Vaccination Campaign

The vaccination intervention was organized by a tripartite partnership comprised of the Swiss Tropical and Public Health (Swiss TPH) Institute, the Centre de Support en Santé Internationale (CSSI), and the Institut de Recherches en Elevage pour le Développement (IRED). Selected members of these three institutions formed the supervisory and technical committees. District and quarter chiefs were invited to an information workshop prior to the campaign and were actively involved in notifying the public and planning the progression. The campaign was launched in both years by the minister of livestock at the World Rabies Day celebrations on 28th September. Vaccinators were recruited among local animal health workers and veterinarians and were trained on animal handling, vaccination, and registration techniques. The campaign was advertised prior to the start through posters distributed by the responsible administrative officers. During the campaign, radio and loud speaker announcements informed the public on the progression and location of the vaccination posts. Loudspeakers were also used to inform the populations close to the respective posts on the vaccination days. Both years, the vaccination campaign lasted 13 weeks, progressing from district to district. Vaccination days were held Friday to Sunday. Each Monday and Tuesday after vaccination in a given zone, data on the coverage level were collected through a household study and counting of dogs in the street on random transects. The data sets where combined in a Bayesian model that estimated total dog population, percent of ownerless dogs, and overall coverage level. A real-time preliminary analysis done in the field guided the campaign progression. A detailed coverage analysis was done after the campaign period each year. The detailed methodology of this analysis is published elsewhere (25).

Wednesday and Thursdays were used for planning and meeting with authorities in the upcoming vaccination zone. Locations of vaccination posts were defined in agreement with the community authorities and were usually close to the house of a block chief, who provided tables and chairs. GPS data on the location of each vaccination post were collected.

The campaign was funded in equal parts by the UBS Optimus Foundation (material, vaccine, and research cost) and the ministry of livestock in Chad (logistics and salary). Based on the dog to human ratio estimated during a dog demographic survey in 2001 and extrapolated to 2012, the required vaccination doses were estimated at 50,000. The Rabisin^®^ vaccine doses were provided by Merial at a cost of 143 FCFA (0.28 USD, exchange rate 2012) per dose. In addition, Merial provided collars to mark vaccinated animals for 150 FCFA (0.29 USD, exchange rate 2012) each and vaccination certificates free of cost. The cold chain was ensured using storage boxes with cooling elements that were delivered together with the vaccine doses. Syringes and needles were procured locally.

The 30 vaccinators were split into 10 teams of 3 people: one responsible for vaccination and collaring and two for registering the animal and completing the vaccination certificate. Three trucks with drivers transported the 10 teams. Each vehicle was attributed to a supervisor responsible for three to four teams. The teams were each equipped with a cooler box for the vaccine and a box containing all necessary material (syringes, needles, registration forms, muzzle, gloves, first aid kit). Every day the material was checked by the supervisor against a control sheet and the performance of the post (number of vaccinated animal by species, working time, vials used) was reported for each team on a data sheet. Supervisors were responsible to replenish posts under their supervision and provide lunch and water. Each supervisor had additional cooler boxes for vaccine and water bottles. Supervisors decided in consultation with the block chiefs and members of the coordination team on the relocation of posts when owner attendance was low. A detailed list of the costs is listed in Table 1 for both years. Material cost is depicted by unit whereas cost for personnel and transport is calculated per day. Each campaign lasted for 37 working days, from October to December 2012 and 2013, respectively. Because the campaign period included two public holidays, vaccinators were paid for 39 days in total in both years. Car rental and fuel cost are calculated for a total of 50 days, including 37 working days and 13 days of sensitization. In 2013, the information campaign was considerably strengthened, which is reflected in the difference between the respective budget lines in 2012 and 2013.

**Table 1:** **List of costs and expenses of dog mass vaccination campaigns in 2012 and 2013**.

	2012			2013		
Cost item	Number of units	Price per unit	Total cost	Number of units	Price per unit	Total cost
**Public sector**						
*Material cost*						
Animal vaccine	18,182	143	2,600,026	22,306	143	3,189,758
Human vaccine	100	21,703	2,170,340	40	21,337	853,480
Collars	18,182	150	2,727,300	22,306	150	3,345,900
Vaccination certificate	Included in vaccine cost					
Syringes and needles	18,182	40	727,280	22,306	40	892,240
Tables and chairs	Provided by block chiefs					
Material transport box	10	20,300	203,000	1	20,300	20,300
Muzzle	14	6,400	89,600	3	6,400	19,200
Rope	10	8,100	81,000	2	1,350	2,700
Registry	10	8,000	80,000	Reused		
Other writing and documentation material (e.g., pen, stamp, paper)	NA	NA	329,750	NA	NA	126,850
Work protection (face mask, coat, first aid kit)	NA	NA	495,348	NA	NA	617,200
Consumables (e.g., garbage bags, gloves)	NA	NA	45,300	NA	NA	8,800
Cooler boxes	17	20,441	347,500	Reused		
*Cost for personnel*						
Training of vaccinators	NA	NA	250,000	NA	NA	314,750
Daily wages vaccinators	39	151,923	5,925,000	39	151,923	5,925,000
Daily wages supervisors	39	10,000	390,000	3	390,000	1,170,000
Daily wages driver (vaccination and sensitization)	50	11,700	585,000	50	13,860	693,000
Fees for local responsibles (district chiefs and block chiefs)	37	51,216	1,895,000	37	47,108	1,743,000
Lunch provisions (per day)	37	42,791	1,583,250	37	50,372	1,863,750
*Transport cost*						
Transport (car rental and maintenance)	50	9,900	495,000	50	8,308	415,383
Daily fuel cost	50	36,532	1,826,576	50	40,896	2,044,824
*Sensitization*						
Information workshop for town authorities	NA	NA	1,172,000	NA	NA	1,296,000
T-shirts, hats, and banners	315	6,746	2,125,000	Reused		
Posters	1,000	719	719,000	1,000	1,114	1,114,000
Leaflets	5,000	204	1,020,000	5,000	163	815,000
Radio announcements	39	25,000	975,000	39	47,821	1,865,000
Loudspeaker	3	20,000	60,000	Reused		
Poster distribution and cost for loudspeaker campaign (per day)	20	12,040	240,800	52	15,731	818,000
*Admin and communication cost*						
Coordination cost	NA	NA	1,365,000	NA	NA	1,215,000
Administrative cost	NA	NA	200,000	NA	NA	100,000
Communication supervisor (per person)	3	190,000	570,000	3	195,000	585,000
Communication coordination (per person)	3	131,667	395,000	3	130,000	390,000
Other cost	1	353,750	353,750	1	5,000	5,000
Total public sector			32,041,820			31,449,135
Mean public cost per dog vaccinated	18,182		1,762	22,306		1,410
**Private sector**						
Lost working time (60 min, 327 CFA)	18,182	327	5,945,514	22,306	327	7,294,062
Transport to vaccination post	18,182	650	11,818,300	22,306	650	14,498,900
Total private sector			17,763,814			21,792,962
Societal cost of the vaccination campaign			49,805,634			53,242,097
Overall cost in USD^a^			98,715			110,747
Cost per dog vaccinated in FCFA			2,739.28			2,387
Cost per dog vaccinated in USD			5.43			4.96

*^a^1 USD = 504.54 FCFA (October 2012); 480.75 FCFA (October 2013)*.

The overall public cost of the vaccination campaign includes material cost, the cost for personnel and transport, as well as cost for the sensitization campaign. In addition to these public costs, the cost of the private sector is considered. These include dog owner expenditures for transportation to the vaccination post and loss of work time. The average waiting time at a post was assumed to be 1 h, valued at 327 FCFA (0.6 USD, exchange rate 2012) based on monthly per capita income in Chad of 52,325 FCFA (104 USD, exchange rate 2012) (26). For transport, the mean cost of 650 FCFA (1.3 USD, exchange rate 2012) was assumed, which corresponds to the price of one liter of fuel. The sum of public and private cost forms the societal costs.

### Epidemiological Monitoring

To assess effectiveness of the intervention, the vaccination campaign was accompanied by an epidemiological study on the incidence of human exposure to animal bites and a study on the incidence of dog rabies cases in N’Djamena.

Data on bite exposure was collected in collaboration with selected health facilities, including public health centers, hospitals, pharmacies, private medical clinics, and a few veterinary practices. Overall 91 facilities were contacted, with 61 completing at least one questionnaire during the study period, from June 2012 to December 2014. The facilities were visited by study members at least once a week to collect completed questionnaires. Collected data included basic information about the bite victim (sex, age, address), the status of the animal (species, vaccination history, alive/deceased), bite history, severity of the wound(s) inflicted, and the treatment recommendation. Data were double entered into Access^®^ databases by the data management team at CSSI. The analysis was done with Stata/IC™ 14. During the data analysis, it was observed that recommendation for PEP made by health personnel was based on the severity of the bite wound rather than on the status of the biting animal. This meant that reported numbers of PEP could not be used as a proxy for human rabies exposure for the DALY calculation nor to estimate the actual number of PEP needed. Therefore, a dummy variable was assigned to each reported case defining victim rabies exposure risk according to fate and vaccination status of the biting animal: (1) high risk exposure (PEP definitely needed for the bite victim) was defined when the animal had been killed, had died, or was missing following the attack, regardless of reported vaccination status; (2) moderate exposure risk (PEP need depending on the observation result) was attributed to bites inflicted by animals with unknown, outdated, or no vaccination history which were alive and could be placed under observation; (3) bites inflicted by a confirmed vaccinated animal which was alive and under observation were not considered as an exposure to rabies (no PEP needed).

These exposure risk categories were used for DALY calculations and to estimate actual number of PEP needed (as opposed to reported number of PEP). In addition to bite cases, information on the cost of human anti rabies vaccine and the vaccination schedule prescribed for PEP was collected during the health facility based study.

The most commonly used protocol was the Essen 5 dose scheme. Other protocols, applied rarely, were the Essen 4 dose scheme and the Zagreb protocol. The details of the protocols are described in Table 2.

**Table 2:** **The three different post-exposure prophylaxis protocols used in N’Djamena [table adapted from Hampson et al. (17)]**.

Protocol	Number of clinical visits	Days of injection after exposure	Number of injections per day	Overall vaccine quantity needed (ml)	Administration pathway	Approved by
Essen 5 doses	5	0,3,7,14,28	1,1,1,1,1	5^a^	IM	WHO (1992)
Essen 4 doses	4	0,3,7,14	1,1,1,1	4^a^	IM	ACIP^b^ (2009)
Zagreb	3	0,7,21	2,1,1	2^a^	IM	WHO (1992)

*^a^Calculated on the basis of 0.5 ml per dose*.

*^b^Advisory Committee on Immunization Practices*.

In parallel to the health facility study, the results of rabies diagnostic tests routinely performed at the IRED laboratory on suspect animals were collected. The observed percentage of rabies positive dogs among all dogs tested was used as a baseline for the probability of an exposure being inflicted by a rabid dog.

### Cost Comparison

The evidence collected on cost of dog vaccination and PEP, together with the epidemiological background information, allowed for evaluation of different control scenarios in regard to their comparative cost effectiveness. The three different scenarios compared were (1) cost of PEP alone, (2) cost of PEP and dog mass vaccination intervention, without communication between the human health and veterinary sector, and (3) cost of PEP and dog mass vaccination, with maximal communication between human and animal health workers (One Health paradigm). Measures for improvement of communication were not part of the study, so scenario 3 is uniquely hypothetical. Under ideal One Health communication conditions, a veterinarian would automatically be contacted for each bite case reported and cases of unknown vaccination status would become negligible, provided that the dog was owned and an effective registration system was in place.

The overall cost of PEP was calculated following examples from other resource limited countries (17, 27) and included medical and non-medical expenditures. Medical fees were comprised of cost of vaccine multiplied by the doses needed for a given schedule, the cost for syringes and needles (here included in the vaccine cost), and institutional costs (salaries, administration). Non-medical costs were costs accrued by victims, including transport and lost working time similar to private costs for vaccination of dogs.

For the calculation of PEP cost alone (scenario 1), the monthly number of PEP recommended by the health personnel within the first 6 months of the study period was used as a basis.

Yearly cost for canine vaccination was derived from the cost description of the vaccination campaigns in 2012 and 2013. From 2014 onward, it was assumed that the two campaigns would lead to interruption of transmission, assuming no reintroduction from outside the vaccinated area, and therefore, only a baseline cost for control of reintroduction and emergency vaccination was included. Cost of PEP for scenario 2 was calculated based on the number of PEP recommended by health personnel observed during the study period. From 2015 onward, the mean number of registered PEP in 2013 and 2014 was used as the calculation basis.

Cost for the hypothetical scenario 3 was calculated by summing the dog vaccination cost as used in scenario 2 and the yearly actual number of PEP needed based on the exposure risk variable defined described above. It was assumed that maximal One Health communication would lead to better decision-making in regard to need of PEP and that there would be fewer animals with unknown vaccination status. Based on the coverage rate achieved during the vaccination campaign (25), 70% of the registered bites from dogs with unknown or unconfirmed vaccination were considered as vaccinated and further excluded from the number of exposures in the scenario. In addition it was assumed that in case of a bite inflicted by an unvaccinated dog, victims would initially start treatment, but discontinue if the animal was still alive after the 10-day observation period.

The age distribution of victims exposed to rabies used for the calculation of averted DALY was based on a previous study on animal rabies cases in N’Djamena (28) reporting proportion of age groups among victims of rabies positive dogs as follows: age 0–5 years 19%; age 5–15 years 36%, and age above 15 years 45%.

For the estimation of averted life years lost by a given scenario, it was assumed that in the absence of PEP, rabies exposure would lead to death in 19% of cases (29). It was further assumed that 66% of suspect exposures are inflicted by a rabid animal. This assumption is based on the proportion of animals tested positive among all animals sent for rabies diagnosis to IRED during the study period.

Because clinical rabies inevitably leads to death within days, only averted life years lost were considered without any adjustment for disability (30). We used the standard formula described by Murray within the model life-table West Level 26 (31).

The discount rate used was 4%. The parameter for the age weight function (*b*) utilized was 0.04 and the constant (*C*) was set at 0.1658. The disability weight function was defined as 1.

Cost efficiency of PEP alone was calculated as discounted cumulative cost of baseline PEP number before the start of the mass vaccination campaigns divided by the cumulative number of DALY averted. Cost efficiency of the mass vaccination campaign and PEP together was calculated from the cumulative discounted cost of canine vaccination and number of PEP registered during the intervention period divided by the difference between the cumulative number of DALY averted by PEP alone and the cumulative number of DALY averted by vaccination of dogs.

### Ethical Consideration

This study was authorized by the ministry for higher education and scientific research (Ministère de l’Enseignement Supérieur et de la Recherche Scientifique) under the document number N°012/PR/PM/MES/SG/DGESRSFP/DRST/012 on May 31, 2012.

The Mayoral office of N’Djamena was informed about the study and gave consent. All personnel involved in immunization of dogs were vaccinated against rabies before participating.

## Results

During the vaccination campaign, a total of 18,182 dogs were vaccinated in 2012 and 22,306 in 2013. The analysis revealed an overall coverage of 71% in both years. On the district level, observed coverage varied widely ranging from 33 to 86% depending on the cultural and socioeconomic background of the area. The dog population of N’Djamena was estimated to be around 30,000 of which only 14% are ownerless (data from 2013). The intervention led to a considerable drop in dog rabies incidence from 0.7/1,000 in 2012 to 0.07/1,000 in 2014. Detailed coverage analysis and epidemiological data are presented elsewhere (25).

The total cost for the 2012 campaign was 98,715 USD (49,805,634 FCFA), and in 2013 the cost was 110,747 USD (53,242,097 FCFA). Expressed in cost per dog vaccinated, 5.43 USD were spent in 2012 and 4.96 USD in 2013 (cats and primates were excluded). The difference between the 2 years was due to higher expenses for sensitization to boost the vaccination coverage. The success of this intensified information campaign is reflected in the higher number of dogs vaccinated in 2013, which increased the overall cost but lowered the cost per animal vaccinated. In both years, the public cost represented roughly 2/3 of the societal cost (64% in 2012 and 59% in 2013).

Over the 2½-year study period, 1,203 questionnaires on bite victims were collected, of which 1,143 matched the inclusion criteria (victim was from N’Djamena, bite inflicted by a mammal species). All recorded incidences were category III exposure, with 902 (79%) inflicted by a dog, 56 (5%) by a cat, and 15 (1%) by a primate, while in 170 (15%) the species was not specified.

**Figure 1:**
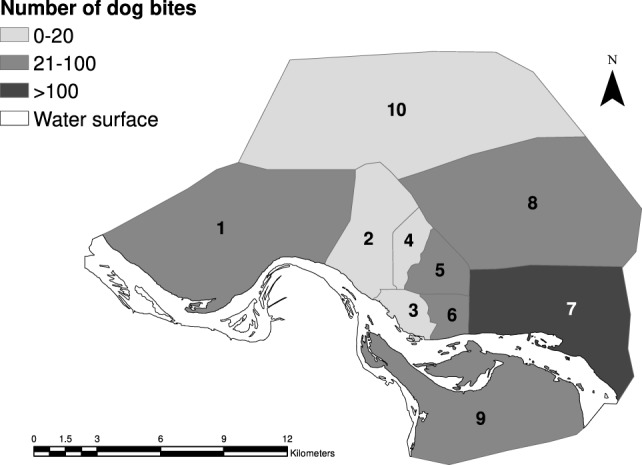
**Distribution of total number of bite cases reported for the 10 different districts of N’Djamena over the whole study period**.

Figure 1 shows the distribution of bite cases by district in N’Djamena. The distribution reflects the difference in dog to human ratio observed during the vaccination campaign (25).

**Figure 2:**
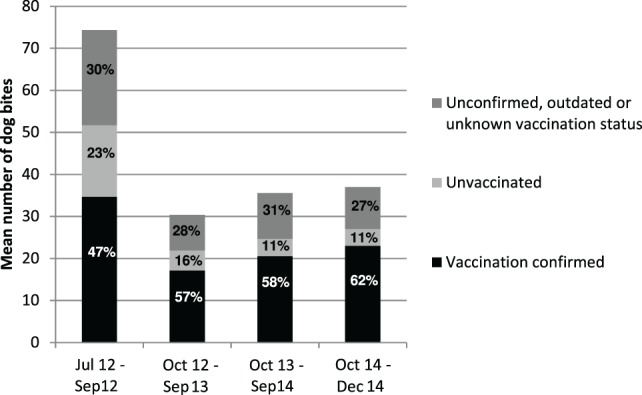
**Mean number of dog bites inflicted per time period and proportion of vaccination status categories**. July to September 2012 corresponds to the pre-vaccination campaign period. June 2012 was excluded due to very low overall number of cases reported.

The highest proportion of bite exposure (42%) was reported in the age group of children younger than 13 years. Overall, 46% of biting animals had a confirmed vaccination status. The vaccination campaign only slightly (10%) increased the number of confirmed vaccinated animals over the period of the mass vaccination intervention (Figure 2).

In 99% of PEP recommendations, the scheme applied was the Essen 5 dose regimen, while the remaining PEP treatments followed the Essen 4 dose or Zhagreb regimen. As the study did not include a follow up of the victims, information on completeness and success of the treatment was not collected. Details on the three different treatment schemes are presented in Table 2. Because the number of Essen 4 dose and Zagreb regimens recommended was negligible, only the cost of the Essen 5 dose regimen was considered for the cost calculation. It was assumed that all victims underwent wound cleaning as recommended by WHO (washing with soap and water for 15 min) (1) and that 40% of victims were accompanied by a parent. One completed course of PEP, therefore, incurred a cost to society of 198 USD (97,512 FCFA) (Table 3).

**Table 3:** **Cost calculation for post-exposure prophylaxis treatment**.

Cost item for a complete five doses Essen protocol	Cost in FCFA	Cost in USD^a^	Unit basis
Vaccine cost (5 doses)	55,000	111.79	Per person
Cost for technician	3,307	6.72	Per person
Cost for syringes and needle	987	2.01	Per treatment
Tetanus vaccine (1 dose)	4,000	8.13	Per person
Antibiotics and anti-inflammatories	11,385	23.14	Per treatment
Water	36	0.07	Per person
Antiseptic	197	0.40	Per person
Lost work time^b^	10,000	20.33	Per treatment
Transport cost^c^	12,600	25.61	Per treatment

Total	97,512	198.20	Per treatment

*Rabies immunoglobulin is not included because of unavailability in Chad*.

*^a^Exchange rate 1 USD = 492 FCFA*.

*^b^40% of exposed victims are accompanied*.

*^c^Expenses for accommodation not included*.

In total, 455 (38%) victims were recommended to follow PEP treatment over the study period. In 202 of these cases, no rabies risk was identified according to the animal status. Conversely, PEP was not recommended in 36 cases where the animal status was defined as high risk and in 289 cases defined as moderate risk during the analysis phase. This indicated that in many cases the recommendation of health personnel was not appropriate. Contact with a veterinary structure was reported in only one-third of overall bite cases (*n* = 349, 30%), and this number might be even lower due to misrepresentation. Using the example of reporting to IRED, it was mentioned in 144 cases (15%) that the animal was brought to the rabies laboratory. However, this number did not reflect the actual registered diagnostic requests at the rabies diagnostic facility over the same period of time. Reflecting this lack of communication between the veterinary and the human health sector, the drop in animal rabies cases induced by the mass vaccination campaign did not lead to a parallel reduction of PEP use.

**Figure 3:**
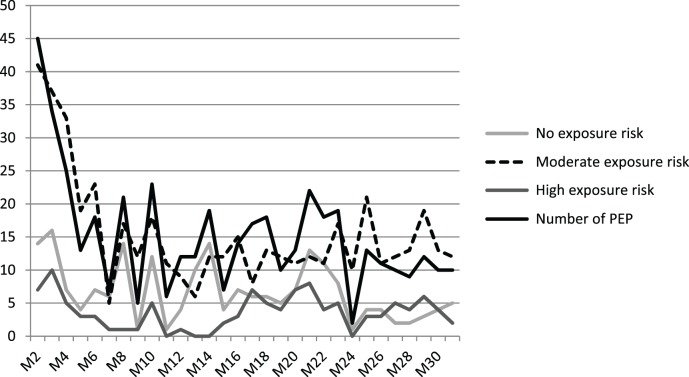
**Number of post-exposure prophylaxis (PEP) recommended per month compared to the number of monthly cases per exposure risk group**.

The amount of PEP used compared to the number of cases within the different risk exposure groups is shown in Figure 3. Stratified by intervention year, a total of 284 PEP recommendations were estimated in 2012, while 164 were observed in 2013 and 149 were registered in 2014. It was assumed that without dog mass vaccination, the demand would have remained at the same level as in 2012, and therefore, PEP numbers from 2012 were used as a baseline for the extrapolation of PEP cases hypothetically occurring from 2013 onward in scenario 1 (Table 4). For scenario 2, the actual reported numbers of PEP recommendations were applied to the respective year of the study period. As no data were available from 2015 onward, the mean number of PEP observed after the vaccination campaign (2013 and 2014) was applied to the following years (2015–2030) (Table 4). For the extrapolations, uncertainty was not accounted for in our recorded data for the sake of clarity.

**Table 4:** **Description of the principle background for the calculation of crude cost and cost-efficiency by scenario**.

Cost calculation					
Scenario	Cost composition	Calculation basis 2012	Calculation basis 2013	Calculation basis 2014	Calculation 2015 onward
1	Post-exposure prophylaxis (PEP)	Number of recommended PEP registered June to December 12, extrapolated to 12 months	Number of recommended PEP registered June to December 2012, extrapolated to 12 months	Number of recommended PEP registered June to December 2012, extrapolated to 12 months	Number of recommended PEP registered June to December 2012, extrapolated to 12 months

2	PEP and vaccination	number of recommended PEP registered in 2012	number of recommended PEP registered in 2013	number of recommended PEP in 2014	mean number of recommended PEP registered in 2013 and 2014

		Cost of vaccination campaign in 2012	Cost of vaccination campaign in 2013	Estimated yearly flat rate for reintroduction control and small scale emergency vaccination	Estimated yearly flat rate for reintroduction control and small scale emergency vaccination

3	PEP and vaccination	Sum of high risk exposures and 30% of moderate risk exposures registered June to December 2012, extrapolated to 12 months	Full cost for Essen 5 doses PEP for sum of high risk exposures and cost of three doses for 30% of moderate risk exposures registered in 2013	Full cost for Essen 5 doses PEP for sum of high risk exposures and cost of three doses for 30% of moderate risk exposures registered in 2014	Full cost of PEP for mean number of high risk exposures and cost for three doses for 30% of moderate risk exposures registered in 2013 and 2014

		Cost of vaccination campaign in 2012	Cost of vaccination campaign in 2013	Estimated yearly flat rate for reintroduction control and small scale emergency vaccination	Estimated yearly flat rate for reintroduction control and small scale emergency vaccination

**Cost-effectiveness calculation**					

**Scenario**	**Calculation of exposures averted**	**Calculation of disability-adjusted life years (DALY) averted**	**Cumulative cost after 20 years**	**DALY averted after 20 years**	**Cost per DALY averted over 20 years**

1	Number of recommended PEP with high and moderate exposure risk background registered in 2012 and extrapolated to the following years	19% of number of exposures averted multiplied with years of life lost (YLL) according to different age classes	388,515,250 FCFA/770,038 USD	6,372	60,971 FCFA/121 USD

2	Difference of extrapolated number of high and moderate risk exposure cases registered in 2012 and effective exposures (100% of high risk and 70% of moderate risk) registered in 2013 and 2014	19% of number of exposures averted multiplied with YLL according to different age classes	349,001,170 FCFA/691,721 USD	9,055	38,544 FCFA/76 USD

3	Difference of extrapolated number of high and moderate risk exposure cases registered in 2012 and effective exposures (only high risk) registered in 2013 and 2014	19% of number of exposures averted multiplied with YLL according to different age classes	287,226,252 FCFA/569,283 USD	9,055	31,721 FCFA/63 USD

*For all costs, a discount rate of 4% was applied*.

*Exchange rate 2012 applied, 1 USD = 504.54 FCFA*.

Because PEP recommendations and exposure risk did not correspond, the real number of PEP needed to prevent 100% of human deaths from rabies would be much higher than actual reported PEP use. In 2012, 374 bite cases of high and moderate exposure risk occurred. In 176 of these cases, PEP was not recommended and assumed to not be administered. Based on the exposure risk cases observed from the health facility data, the cost of effective rabies prevention in humans by use of PEP alone would require an investment of 36,469,488 FCFA each year as opposed to the observed yearly investment of 27,693,408 FCFA.

As a result of the vaccination campaign, the observed exposure (assuming 70% vaccination coverage) declined in 2013 to 102 cases and in 2014 a total of 135 exposure cases were observed. This corresponds to a yearly mean of 119 exposures for which a rabies infection in the biting animal could not be excluded and which would, therefore, require PEP, despite the mass dog vaccination, to ensure that 100% of human rabies cases are prevented. Compared to the mean of 156 PEP recommendations which still occurred after the vaccination campaign, the number of PEP would at least be reduced by 24% (*n* = 37) with better One Health communication.

**Figure 4:**
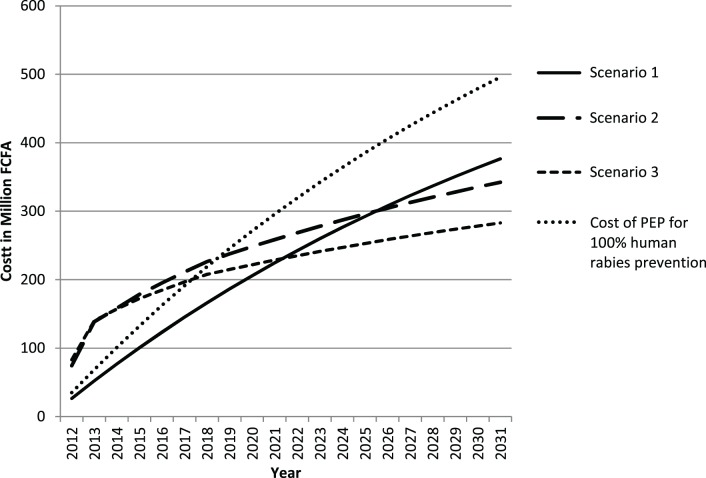
**Display of the cost trend of the three different rabies control scenarios**.

The background for the cost analysis and the results of the cost comparison of the three different scenarios are presented in Table 4 and Figure 4.

Intervention in the animal reservoir shows a clear advantage over prevention measures solely on the human medical side. The break-even point of dog vaccination and PEP with sole use of PEP is forecast about 15 years after the start of intervention. If maximum One Health communication was achieved in addition to dog vaccination, the cost even point is reached only 10 years after the start of the intervention. This is due to reduction in inappropriate use of PEP (dog confirmed vaccinated and in good health during the observation period), which leads to lower human vaccine cost for scenario 3 compared to scenario 2.

The advantage of investment into the veterinary sector for the control of rabies in humans became even more striking when cost efficiency per DALY averted was compared. The yearly number of DALY averted with scenario 1 was 454, whereas dog vaccination led to a total of 659 DALY averted each year. This showed that PEP use as currently applied in N’Djamena is costly but does not prevent human rabies cases because the exposure of humans to rabid animals remains, and many bite victims who are in need do not get PEP due to failure to consider the status of the biting animal.

Vaccination of the animal vector was about 30% less costly over a period of 20 years and this cost efficiency would be improved by strengthening communication between animal and human health workers. Overall, yearly number of DALY averted with scenario 2 and 3 are the same, but One Health communication led to a significant reduction of PEP cost compared to dog vaccination alone. In the absence of such communication, the cumulative number of PEP prevented by the vaccination intervention after 20 years was only 2,423 as compared to 4,057 doses of PEP prevented with scenario 3. This difference led to a slightly higher cost effectiveness of scenario 3 compared to scenario 2 (Table 4; Figure 4).

Because the observed PEP recommendations in this study were not able to prevent human rabies deaths, we calculated the cost for optimal use of PEP in N’Djamena, defined as numbers of PEP correspond to the number of observed high and moderate exposure risk cases. Compared to these estimated costs for prevention of 100% of human rabies deaths by PEP only, scenario 3 would be advantageous after 6 years and scenario 2 after 8 years.

## Discussion

The cost analysis of the vaccination campaigns in 2012 and 2013 are based on a previous description of the cost of a pilot vaccination campaign in the same town (19). Compared to the former campaign in 2003, the costs per dog vaccinated almost doubled due to higher personnel cost, which reflects the economic development over the past 10 years in Chad.

Our costs observed were considerably higher than described by Shwiff et al. (18), where a mean of 1.55 USD was grossly estimated for Africa, Asia, and Latin America. As the success of a rabies vaccination campaign depends on the coverage achieved, the goal is to maximize the number of animals vaccinated per dollar invested. Lower cost at the expense of lower coverage might lead to lower cost effectiveness because of ineffective control of the disease. This was illustrated in Tanzania where a study compared cost effectiveness for different coverage levels (16). As coverage depends heavily on accessibility of dogs and accessibility is in turn defined by the socio-demographic, cultural, and economic background of the human population, costs vary greatly between different regions (32). Even across a limited area, such as the town of N’Djamena, costs vary significantly between different contexts (25).

Although the total number of vaccination days remained the same in 2013 compared to 2012 and higher costs were observed in the second year due to reinforcement of sensitization, the cost per dog vaccinated was lower in 2013. This highlights that the number of dogs vaccinated per day is an important factor for cost effectiveness of a campaign and that investment to enhance accessibility is beneficial.

Ongoing surveillance showed a reduction of weekly animal rabies incidence. Animal rabies surveillance in our study was based on passive reporting of suspected cases. A study in Tanzania suggests that a passive surveillance system is only able to detect 1% of actual rabies cases (33), and Townsend et al. estimate that only a detection success of 10% can prove the absence of animal rabies within the time period of 2 years (34). In our study reporting was boosted by a sensitization campaign, and we also hypothesize that the urban context with high human density leads to a higher detection rate. Therefore, we are confident that we were able to detect at least 10% of dog rabies cases occurring within the city limits.

Despite the reported decline of animal rabies incidence after the vaccination campaigns, PEP demand did not decrease to an equal extent. During the analysis phase, it became evident that health personnel judged the rabies risk according to the severity of the wound inflicted and only rarely considered the animal background. In consequence, PEP was sometimes provided without real indication, causing an unnecessary burden for the public sector and for households with low income. Regardless of the investment into animal rabies, the cost effectiveness of PEP could be increased through better communication between veterinarians and human health workers and also through changing from the intramuscular Essen regime to an intradermal protocol (17).

Overall, the predicted break-even point between dog mass vaccination with PEP and PEP alone in the earlier simulation (21) matches the observed time period under a scenario of no reintroduction from outside of town. The main difference to the earlier work is that two campaigns were needed instead of one. Given the suggested interruption of rabies transmission in N’Djamena by the high coverage achieved in both years and the subsequent drop in need for PEP, dog vaccination would have a higher cost effectiveness after 15 years, despite the high investment. This is due in part to the equally high cost for PEP observed in Chad. Even without inclusion of RIG, an entire course of PEP equals the cost of PEP with RIG in South Africa (35) and is considerably higher than in Tanzania (29). This means that if the WHO recommended inclusion of RIG for Category 3 exposures was applied in Chad, canine vaccination would have a much higher cost effectiveness than described in this study. For rural areas where accessibility is geographically very limited, the cost for travel and accommodation would also be higher than that presented here for an urban setting.

However, on the veterinary side, costs could also be higher because some were not included within the calculation presented, for example, costs related to animal observation, surveillance, and diagnostics. Finally, with regard to the DALY, we only considered cost in regard to years of life lost. Rabies also leads to a psychological burden in families of victims and in exposed people who fear contracting the disease (3, 36). This psychological aspect of disability has not been described empirically to date but potentially leads to a productivity loss and higher burden of disease.

Our results show that One Health communication is crucial to get a maximum return on the investment and prevent prevailing unnecessary high PEP cost. The overall number of DALY averted in scenario 2 was equal to scenario 3, but scenario 3 had higher cost-efficiency due to lower investment into PEP. This highlights that dog vaccination together with One Health communication allows for maximal translation of effect on rabies control in the animal sector to cost saving for the public health sector.

One health communication also incurs cost, for example, meeting fees, transport costs, and telephone credit. These costs were not included in the calculation due to absence of reliable data. Also, PEP cost calculation for scenario 3 was based on the assumption that, if the biting animal is not vaccinated, an observation period of 10 days applies, during which PEP in all victims is already initiated. This means that for the duration of the observation period 3 doses of vaccine would be needed. Currently, the observation period applied in Chad is 14 days, which requires 4 doses before discontinuing the unnecessary treatment.

The cost efficiency calculation was based on the assumption that canine rabies transmission can be interrupted in N’Djamena by two vaccination rounds. Thus after 2 years, only the costs for prevention of reintroduction are incurred. Disease modeling and phylodynamic analysis of the epidemiological and molecular data collected during the study period suggest that interruption was achieved but that rabies was re-introduced from outside the relatively small (254 km^2^) vaccination area (37).

Data from ongoing routine diagnostics at IRED also show that without control at the town border, rapid reintroduction into the city occurs. The epidemiological pressure from the rural to urban areas is also described for Bangui and its surroundings (38). Therefore, sustainable control can only be achieved with either stringent reintroduction control for rabies free areas requiring movement restrictions on dogs or large scale national campaigns. A preliminary budget estimate for a Chadian national dog rabies vaccination campaign suggests costs between 1.9 and 4.7 million Euros, depending on the number of dogs vaccinated per day per vaccination post and the overall duration (22). Despite several limitations and assumptions, our study proves the financial advantage of investment into dog mass vaccination for prevention of human rabies, identifies the need for better communication between the human and animal health sectors to improve cost effectiveness of interventions in the animal reservoir, and highlights the urgency for large scale control of animal rabies in Chad.

## Conclusion

Despite the high initial cost for mass vaccination, the advantage of investment into rabies control in the host species is evident. Our results clearly show that canine mass vaccination has a higher cost effectiveness per DALY averted than PEP alone and is less costly over a period of 15–20 years. The study successfully demonstrates the added value of a One Health approach in zoonotic disease control. PEP remains the main prevention strategy to avert rabies deaths but compared to animal vaccination, it is not cost effective and does not lead to reduction and elimination of human rabies cases.

## Author Contributions

RM: data collection, analysis, and writing of the paper. ML: data collection, analysis, and writing of the paper. AO: data collection and analysis. KN: data collection. BK: data collection. LO: analysis and writing of the paper. SS: analysis and writing of the paper. IA: veterinary supervision of the study. DM: medical supervision of the study. JZ: study design, analysis, and paper writing.

## Conflict of Interest Statement

The authors declare that the research was conducted in the absence of any commercial or financial relationships that could be construed as a potential conflict of interest.
